# Supplementary material for: Aneuploid senescent cells activate NF‐κB to promote their immune clearance by NK cells
Source: EMBO Rep. 2021 Jun 8;22(8):e52032. doi: 10.15252/embr.202052032 (PMC8339690; doi:10.15252/embr.202052032)
Supplement: Supplementary file 1 — Expanded View Figures PDF [file EMBR-22-e52032-s001.pdf]

Expanded View Figures

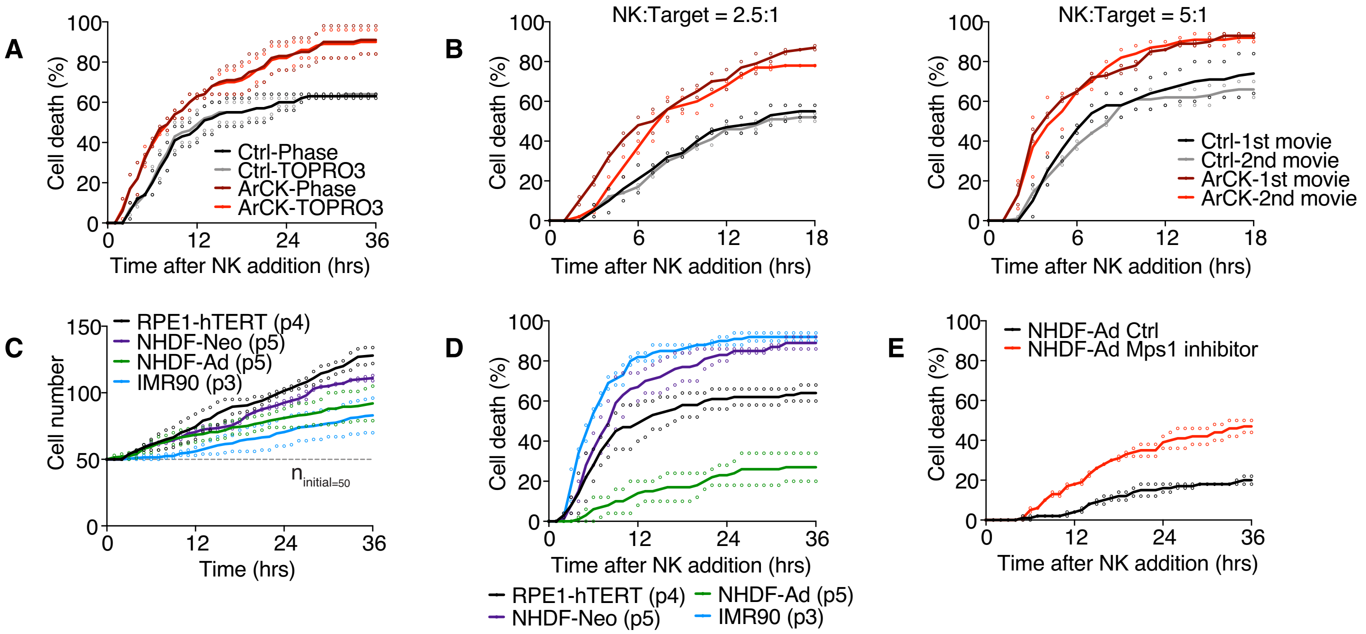

Figure EV1. Characterization of the NK cell killing assay.

A Side-by-side comparison analyzing NK cell-mediated killing on euploid control or ArCK cells by phase contrast image (Phase) or TO-PRO3 signal. Cells were cultured as described in Fig 1A. Statistical analyses were performed as in Fig 1C. Individual points and mean were presented; $n = 2$ biological replicates. Ctrl-Phase vs. Ctrl-TOPRO3, $P = 0.89$, *n.s.*; ArCK-Phase vs. ArCK-TOPRO3, $P = 0.89$, *n.s.*; KS test.

B Measurement of NK cell-mediated killing of ArCK cells in two consecutive 18-h time lapse experiments. After the first 18 h of the analysis, the cell suspension was collected and co-cultured with a second set of target cells. NK cell-mediated killing was measured in the first (black and dark red curves) and the second (gray and light red curves) 18-h time lapse and plotted on the same graph. The killing assay was performed at a NK cell-to-target cell ratio of 2.5:1 (left panel) and 5:1 (right panel); $n = 2$ biological replicates. NK:Target = 2.5:1, Ctrl-1st movie vs. Ctrl-2nd movie, $P = 1.00$, *n.s.*; ArCK-1st movie vs. ArCK-2nd movie, $P = 0.21$, *n.s.*; NK: Target = 5:1, Ctrl-1st movie vs. Ctrl-2nd movie, $P = 0.28$, *n.s.*; ArCK-1st movie vs. ArCK-2nd movie, $P = 0.97$, *n.s.*; KS test.

C Cell proliferation measurements in the absence of NK cells. RPE1-hTERT (passage 4), human normal neonatal or adult human dermal fibroblasts (NHDF-Neo, passage 5 or NHDF-Ad, passage 5), and human embryonic lung fibroblast (IMR90, passage 3) were plated side by side in NK cell medium, and cell proliferation rate was recorded using live cell imaging as described in Fig 1D. The dashed line indicates the starting cell number ($n_{initial} = 50$). Dot plot of individual data points and mean is shown; $n = 2$ biological replicates.

D NK cell-mediated cytotoxicity across different cell types. The killing of RPE1-hTERT, human normal neonatal or adult human dermal fibroblasts (NHDF-Neo or NHDF-Ad), and human embryonic lung fibroblast (IMR90) were measured as described in Fig 1 using a NK cell-to-target cell ratio of 2.5–1; $n = 2$ biological replicates. Individual data and mean are shown.

E Human normal adult dermal fibroblasts (NHDF-Ad) were treated with either DMSO or the Mps1 inhibitor reversine (500 nM) for 24 h. Drugs were washed out, and NK cell-mediated killing was compared between DMSO-treated (NHDF-Ad Ctrl) and Mps1 inhibitor-treated (NHDF-Ad Mps1 inhibitor) cells as described in Fig 1C; $n = 2$ biological replicates. NHDF-Ad Ctrl vs. NHDF-Ad Mps1 inhibitor, $P = 0.001$; KS test.

© 2021 The Authors

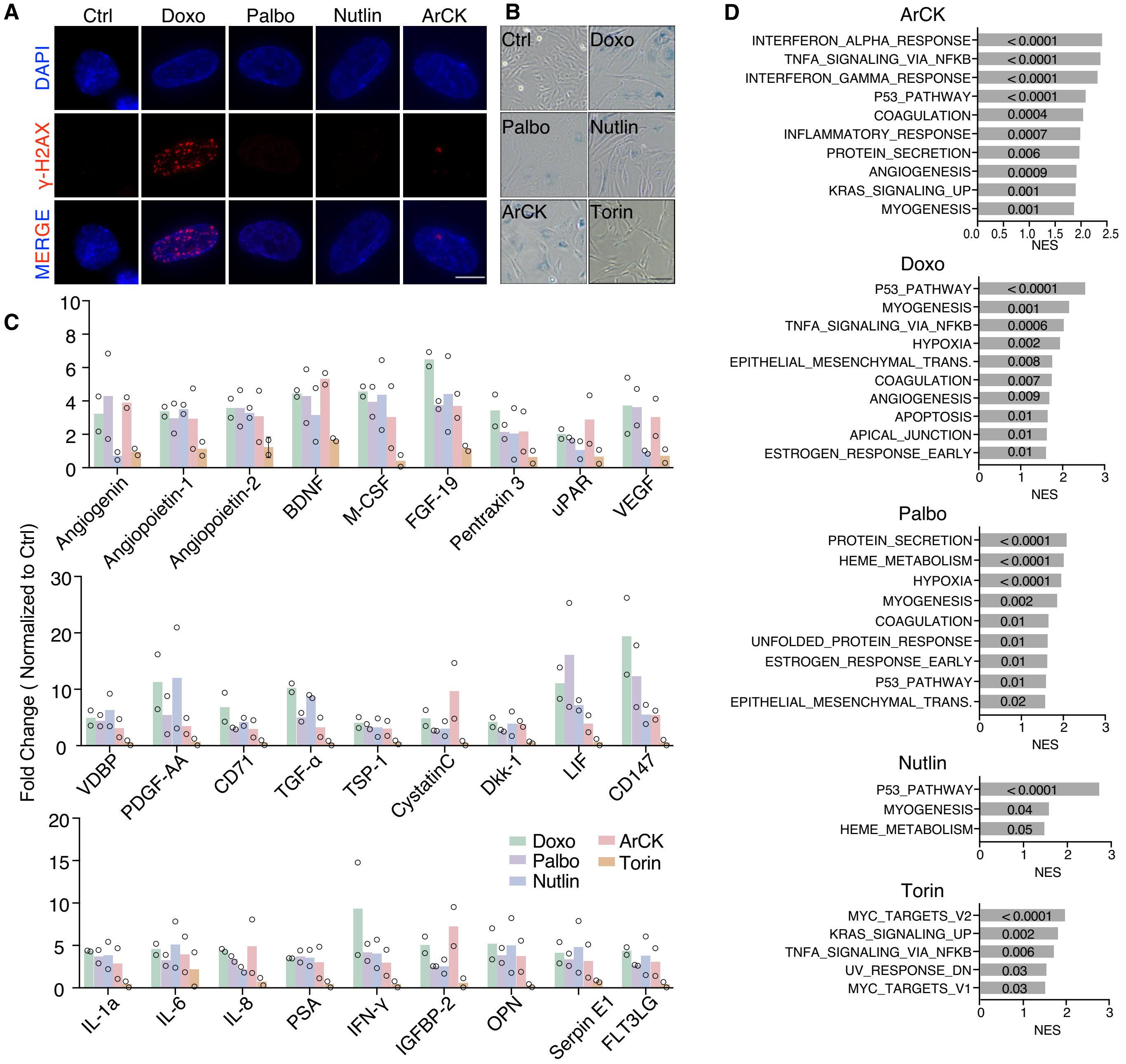

Figure EV2. Characterization of aneuploid- and G1-arrested cells.

- A Representative images of γ-H2AX staining in the indicated samples. γ-H2AX is in red and DNA in blue. Scale bar 10 μm.
- B Representative image of senescence-associated β-galactosidase staining in the indicated samples. Scale bar 100 μm.
- C Analysis of all cytokines secreted by the indicated cells. Cytokine levels are shown as fold change in euploid control cells; $n = 2$ biological replicates. Individual values and mean are plotted.
- D Gene set enrichment analysis (GSEA) for doxorubicin-treated (Doxo), palbociclib-treated (Palbo), nutlin3-treated (Nutlin), torin1-treated (Torin) and ArCK cells relative to euploid proliferating control cells. Only the top 10 ranked hallmarks are presented in Doxo, Palbo, and ArCK conditions. The normalized enrichment score (NES) is plotted. The numbers on the NES score bar indicate the corresponding *p*-values for each hallmark (*FDR q value* ≤ 0.05).

 © 2021 The Authors

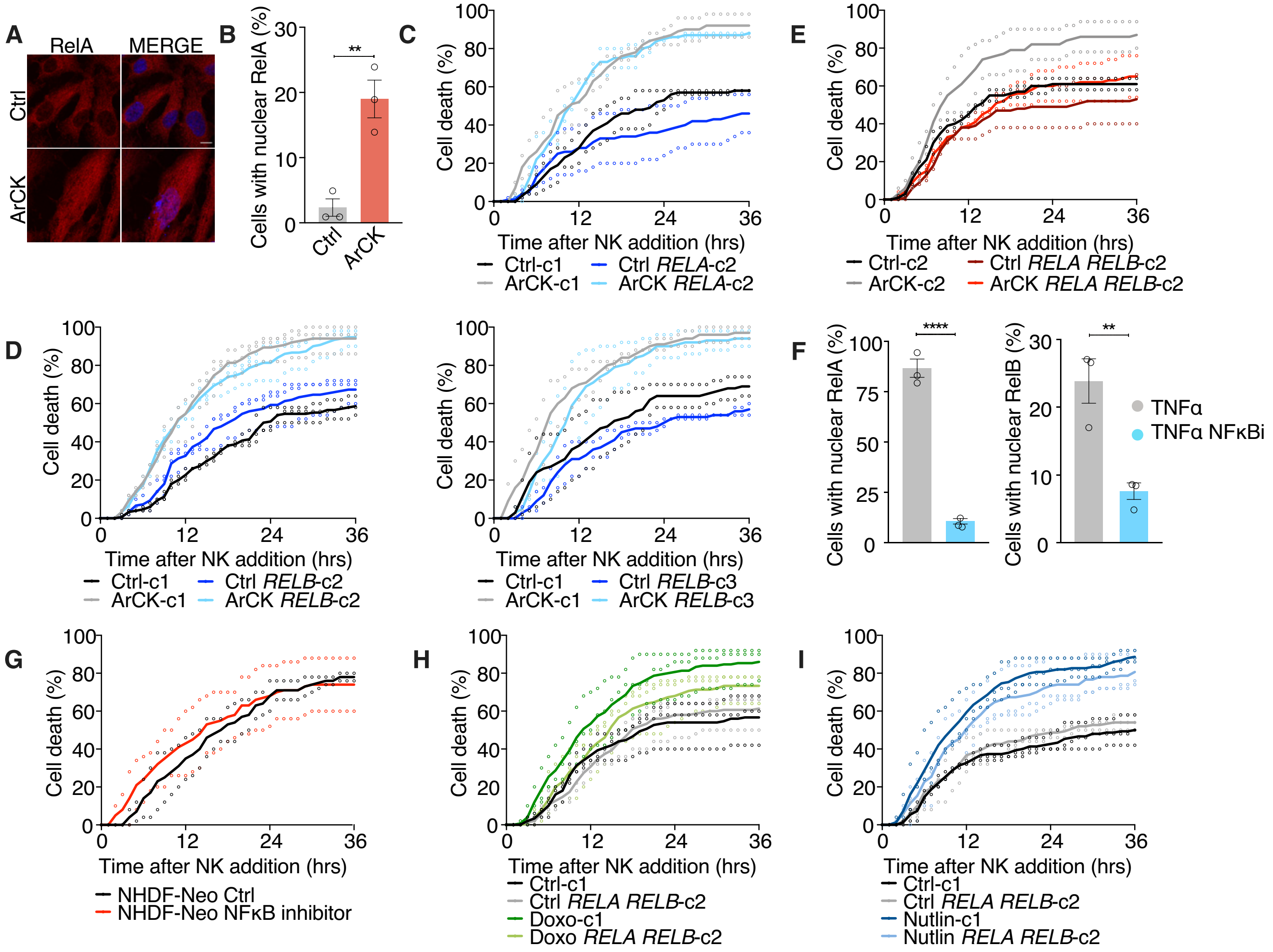

Figure EV3. NF-κB pathway contributes to NK cell-mediated killing in ArCK cells.

A, B Representative images (A) and quantification (B) of RelA nuclear translocation signal in ArCK and euploid control cells. At least 86 cells were analyzed per condition per replicate. $n = 3$ biological replicates; mean ± SEM. $^{**}P = 0.0063$; unpaired t-test. Scale bar 10 μm.

C–E The effect of inactivating *RELA* (C), *RELB* (D), and both *RELA* and *RELB* (E) on NK cell-mediated cytotoxicity in ArCK cells. The experiment was performed as described in Fig 5, except a different single cell clone was used. ArCK-c1 vs. ArCK *RELA*-c2, $P = 0.28$, *n.s.*; ArCK-c1 vs. ArCK *RELB*-c2, $P = 0.72$, *n.s.*; ArCK-c1 vs. ArCK *RELB*-c3, $P = 0.02$; ArCK-c2 vs. ArCK *RELA RELB*-c2, $P = 0.0004$; KS test. The same controls are plotted in (E) and Fig 5E since all three replicates for both *RELA RELB* KO clones were performed side by side at the same time.

F RPE1-hTERT cells were treated with either DMSO or the NF-κB inhibitor BMS-345541 (5 μM) for 48 h. TNF-α (100 ng/ml) was added to cells in both conditions for 1 h prior to cell fixation. RelA and RelB nuclear translocation was quantified and shown. $n = 3$ biological replicates; mean ± SEM. RelA, TNF-α vs. TNF-α NF-κB inhibitor, $^{****}P < 0.0001$; RelB, TNF-α vs. TNF-α NF-κB inhibitor, $^{**}P = 0.0097$; unpaired t-test.

G Human normal neonatal dermal fibroblasts (NHDF-Neo) were treated with either DMSO or the NF-κB inhibitor BMS-345541 (5 μM) for 48 h. The drug was washed out, and NK cell-mediated killing was assessed as described in Fig 1C. Dot plot of individual data points and mean is presented in all of the NK cell killing assays; $n = 2$ biological replicates. NHDF-Neo Ctrl vs. NHDF-Neo NF-κB inhibitor, $P = 0.28$, *n.s.*; KS test.

H, I The effect of inactivating both *RELA* and *RELB* on NK cell-mediated cytotoxicity in 7-day doxorubicin (H) and nutlin3-treated (I) cells. The experiment was performed as described in Fig 5, except a different single cell clone was used. $n = 3$ biological replicates; Doxo-c1 vs. Doxo *RELA RELB*-c2, $P = 0.04$. Nutlin-c1 vs. Nutlin *RELA RELB*-c2, $P = 0.18$, *n.s.*; KS test.

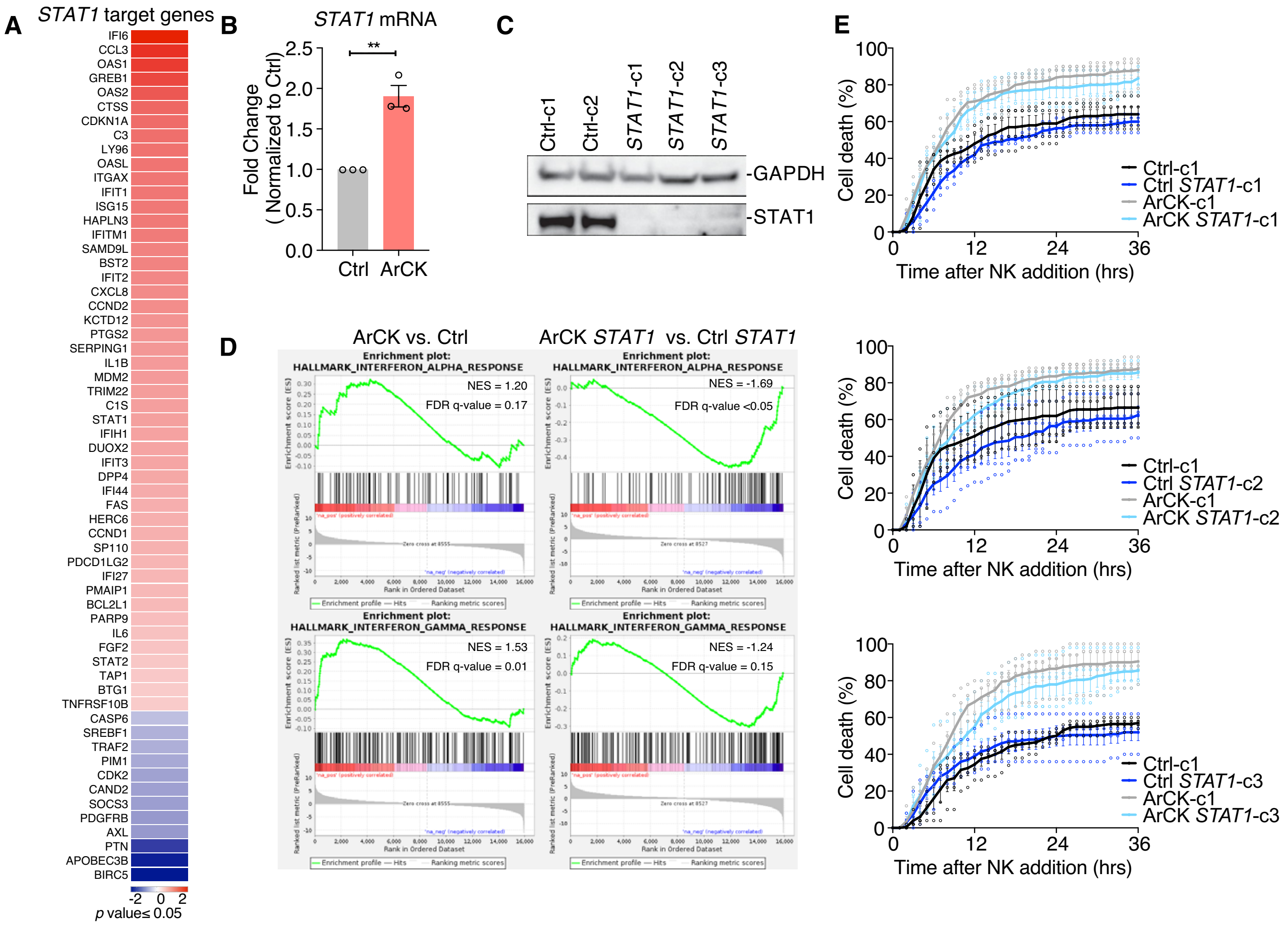

Figure EV4. STAT1-response facilitates immune recognition of ArCK cells.

A Significantly differentially expressed *STAT1* target genes in ArCK cells compared with euploid control cells were identified by ingenuity pathway analysis based on RNA sequencing data. (Log$_2$ fold change, *p*-value $\leq$ 0.05).

B Measurement of *STAT1* mRNA levels in ArCK cells normalized to euploid control cells. $n = 3$ biological replicates; mean ± SEM. $^{**}P = 0.0025$; unpaired *t*-test.

C Measurement of STAT1 protein levels in *STAT1* KO single cell clones generated in RPE1-hTERT cells.

D GSEA enrichment plot for interferon alpha and interferon gamma hallmarks in ArCK cells generated in either control or *STAT1* KO RPE1-hTERT cells. Two single cell cloned control cell lines and three single cell cloned *STAT1* KO cell lines (shown in C) were used in the RNA-seq analysis.

E ArCK cells lacking *STAT1* were generated as described in the Method section. NK cell-mediated cell death in *STAT1* ArCK cells was compared with control cells which harbored an empty vector. $n = 4$ biological replicates; mean ± SEM. ArCK-c1 vs. ArCK *STAT1*-c1, $P = 0.7$, *n.s.*; ArCK-c1 vs. ArCK *STAT1*-c2, $P = 0.05$, *n.s.*; ArCK-c1 vs. ArCK *STAT1*-c3, $P = 0.14$, *n.s.*; KS test.

 © 2021 The Authors

Figure EV5. Analysis of cGAS-STING and retrotransposon activity in ArCK cells and NF-κB activation in cancer cells following chromosome mis-segregation.

A IRF3 and phospho-IRF3 levels were analyzed by Western blot in euploid proliferating cells (Ctrl), cells 60 h post-reversine treatment (Rev60), or ArCK cells that are either functional for *STING* (lanes 1-3) or lacking the gene (lanes 4–6). RPE1-hTERT cells treated with cGAMP (10 μg/ml) for 24 h were used as a positive control (lanes 7 and 8). To confirm the specificity of the phospho-IRF3-antibody, protein lysates from cGAMP-treated cells were incubated with lambda phosphatase (lane 8).

B ArCK *STING* KO cells were generated, and NK cell-mediated cytotoxicity was compared with control cell lines that harbor an empty vector as described in Fig 1C; $n = 2$ biological replicates. ArCK vs. *STING* ArCK, $P = 0.91$, *n.s.*; KS test.

C Measurement of *DDX58* and *IFIH1* mRNA levels in ArCK cells by RT–qPCR shown as fold change compared with euploid control cells. RPE1-hTERT cells treated with PolyIC (10 μg/ml) for 24 h were used as a positive control. $n = 4$ biological replicates; mean ± SEM. *DDX58*, Ctrl vs. ArCK, ***$P = 0.0001$; Ctrl vs. PolyIC, **$P = 0.0023$; *IFIH1*, Ctrl vs. ArCK, *$P = 0.029$; Ctrl vs. PolyIC, ***$P = 0.0003$; unpaired *t*-test.

D ArCK cells were grown as described in Fig 1A to determine ORF1 protein levels. RPE1-hTERT cells treated with azacitidine (Aza, 5 μM) for 5 days (lane 4) were used as a positive control. ORF1p levels under both long exposure and short exposure were presented. Results were comparable between 2 biological replicates.

E The effect of inhibiting reverse transcriptase activity on NK cell-mediated cytotoxicity in aneuploid cells. ArCK cells were generated as described in Fig 1A and were continuously treated with reverse transcriptase inhibitor 3TC (7.5 μM) following chromosome mis-segregation. Control RPE1-hTERT cells were treated with 3TC for 3 days. 3TC was washed out during the NK cell co-culture assay. $n = 3$ biological replicates; mean ± SEM. ArCK vs. ArCK-3TC, $P < 0.0001$; KS test.

F HCT116 and DLD1 cells were treated with either DMSO (Ctrl) or the Mps1 inhibitor reversine (500 nM) for 48 h (Aneuploid). The percentage of cells with nuclear RelA (left) and nuclear RelB (right) signals were quantified. $n = 3$ biological replicates; mean ± SEM. RelA, HCT116 Ctrl vs. HCT116 Aneuploid, ****$P < 0.0001$; DLD1 Ctrl vs. DLD1 Aneuploid, *$P = 0.017$. RelB, HCT116 Ctrl vs. HCT116 Aneuploid, **$P = 0.004$; DLD1 Ctrl vs. DLD1 Aneuploid, **$P = 0.006$; unpaired *t*-test.

© 2021 The Authors

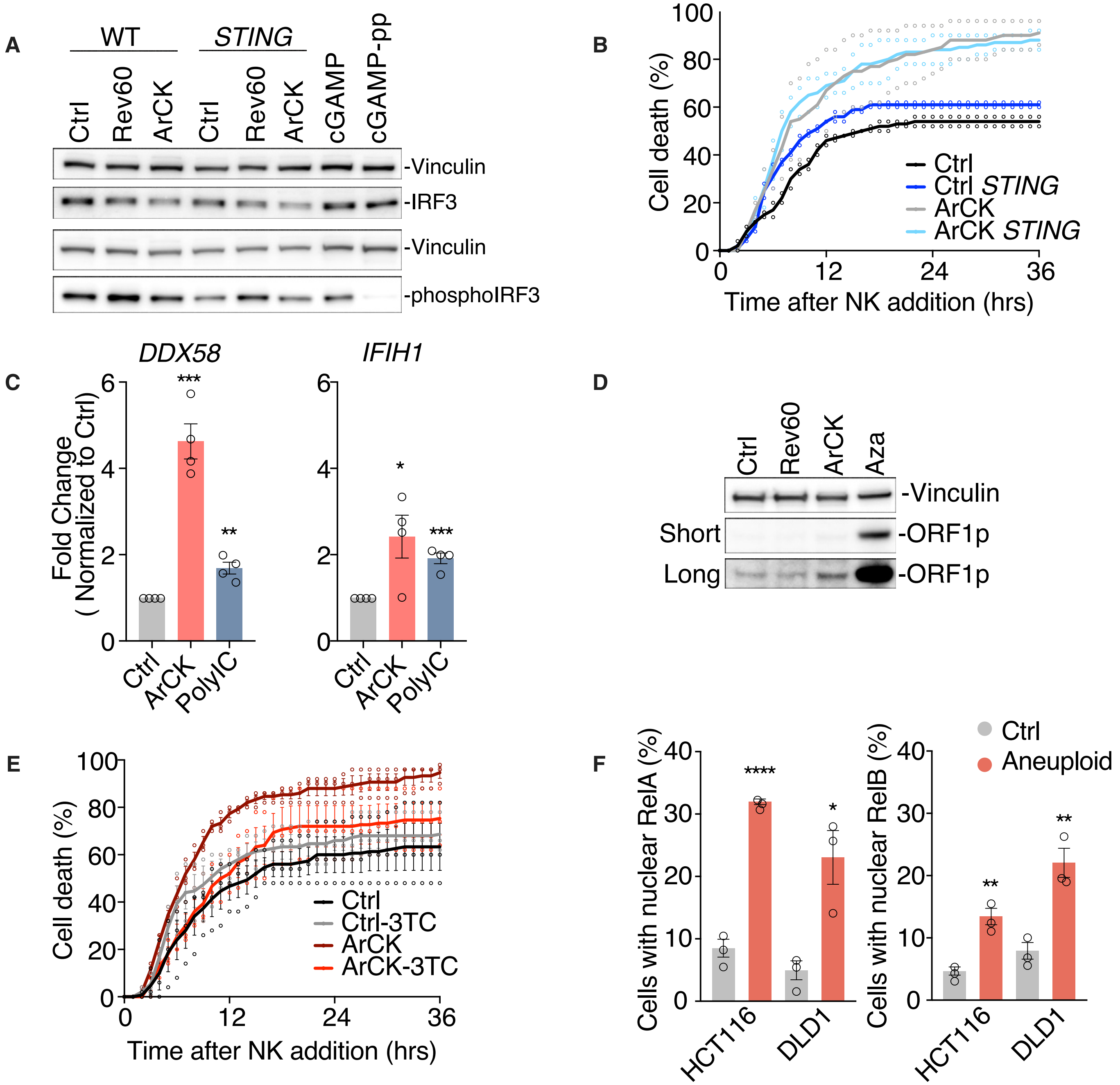

Figure EV5.

 © 2021 The Authors
